# The Average Man Does Not Exist

**DOI:** 10.3390/ijerph19042094

**Published:** 2022-02-13

**Authors:** Johan Molenbroek

**Affiliations:** Faculty of Industrial Design Engineering, Department of Industrial Design, Delft University of Technology, 2628 CE Delft, The Netherlands; j.f.m.molenbroek@tudelft.nl

Public health will improve if the prevention means take care of the variation in human characteristics and do not only consider the average or reference man [1,2]. The average male does not predict the characteristics for an average women. However, still, we create things, spaces and services for 30-year-old white males. We all should know that when we start a research or design project, for example, face recognition, prototyped on white male mostly does not work for black female. When we test a consumer product, a medicine or a service with only adult white males, the results will only be valid for adult while males. Size is currently an easy variable to measure with a 3D scanner, but the budget is always limited, and sometimes it is easier to measure males as opposed to females. This means there is a risk clothing does not fit, especially for people on the extreme ends of the normal distribution, and accidents could be caused, for example, when reaching as a female in a safety suit that is too large. Another aspect is the body temperature of females, which is lower than that of males in the same room, which can be measured with an infrared sensor [2]. Therefore, this means that the 20 degrees Celsius room temperature as a standard might be uncomfortable for females.

Further, regarding senior-proof bicycles, there is a gap between the speed of a usual bike (maybe around 10 km/h) and the speed of an e-bike that can easily be set at a maximum of 25 km/h. In recent years, this gap has resulted in more traffic accidents involving the elderly and e-bikes in the Netherlands. It is easy to describe a policy for local decision makers to organize introduction courses as a prevention measurement for first-time e-bike users and to limit e-bikes to the speed that was used on the old bikes.

A child is not a small adult: the proportions are different. Therefore, you cannot say a child of 100 cm tall has 50% of the arm and leg length of an adult male of 200 cm. Children’s arms and legs are much shorter because they will grow at a later stage.

Obesity is a growing issue in Western countries. Many papers have been written on obesity. Frequent treatments include diets that do not last, or exercises and surgery that help only for a short while. All measurements that are taken in a social context such as within the family or society seem to last longer. Problems still need to be solved in the area of stigmatization of obesity, and medicines are mostly tested on average young males, so females, older people and obese people are often excluded from proper tests [3]. Lastly, public spaces seem to be created for the average man and not for the abovementioned subgroups of our society [1,2,4].

We can talk about handicaps of people, but, in fact, it is better to say society is handicapped because it excludes many subgroups from comfortable usage [3].

## Data Availability

No new data were created or analyzed in this study. Data sharing is not applicable to this article.
